# The Effect of Adding Procalcitonin to the Systemic Inflammatory Response Syndrome (Sirs) and Quick Sepsis-Related Organ Failure Assessment (qSOFA) Scoring System in Predicting Sepsis Mortality

**DOI:** 10.7759/cureus.31740

**Published:** 2022-11-21

**Authors:** Pinak Shah, Shobhit Keswani, Leo Yamaguchi, Kartika Shetty, Elizabeth Benge, Abdul Gader Gheriani, Maycky Tang, Nazanin Sheikhan, Napatkamon Ayutyanont, Andrew Kim, Cristian Valdez, Tony Alarcon

**Affiliations:** 1 Internal Medicine, Mountainview Hospital, Las Vegas, USA; 2 Department of Infectious Diseases, Ochsner Clinic, New Orleans, USA; 3 Internal Medicine, Riverside Community Hospital, Riverside, USA; 4 Clinical Research, Southern Hills Hospital and Medical Center, Las Vegas, USA

**Keywords:** sirs positive + procalcitonin >2 ng/ml (psirs+), qsofa positive + procalcitonin ≤2 ng/ml (pqsofa-), qsofa positive + procalcitonin >2 ng/ml (pqsofa+), sirs positive + procalcitonin ≤2 ng/ml (psirs-), procalcitonin, systemic inflammatory response syndrome (sirs), quick sequential organ failure assessment (qsofa), in-hospital mortality

## Abstract

Objective: The primary objective of this study was to determine if the addition of procalcitonin to the existing systemic inflammatory response syndrome (SIRS) and quick Sepsis-related Organ Failure Assessment (qSOFA) scoring systems could improve the predictability of in-hospital sepsis-related mortality. Secondarily, we sought to determine if the addition of procalcitonin could predict the likelihood of ICU admission and discharge home.

Design: This is a retrospective, single-center, observational study that looked at data from January 1, 2017 to January 1, 2019. Patients were stratified into four groups: SIRS-positive + procalcitonin >2 ng/mL (pSIRS+), SIRS-positive + procalcitonin ≤2 ng/mL (pSIRS-), qSOFA-positive + procalcitonin >2 ng/mL (pqSOFA+), and qSOFA-positive + procalcitonin ≤2 ng/mL (pqSOFA-).

Setting: The study was conducted at a community hospital in Las Vegas, Nevada.

Patients: Patients were included in the study if they were >18 years of age and had hospital admission diagnosis of sepsis with at least one value of procalcitonin level.

Interventions: After patients which met the inclusion criteria, patients were divided into subgroups of SIRS, SIRS + procalcitonin > 2 ng/mL, qSOFA, qSOFA + procalcitonin >2 ng/mL.

Primary outcomes were in-hospital mortality and secondary outcomes were ICU admission, length of stay, and discharge to home.

Results: 933 patients were included in the study with an overall mortality rate of 21.22%, an overall ICU admission rate of 56.15%, and an overall discharge home rate of 29.58%. In those identified with a sepsis-related diagnosis code, pSIRS+ predicted an in-hospital mortality rate of 31.89% compared to pSIRS- 16.15% (P < 0.0001). In regards to qSOFA, the addition of procalcitonin added no statistically significant difference in predicting in-hospital mortality. pSIRS+ patients were found to have an ICU admission rate of 76.16% and a discharge home rate of 19.20% compared to pSIRS- who had 47.40% and 34.90%, respectively (P < 0.0001). Like in our primary outcome, our data for qSOFA was not statistically significant.

Conclusions: Procalcitonin added utility to the SIRS scoring system in predicting sepsis-related in-hospital mortality, ICU admission, and discharge home. Procalcitonin did not add statistically significant benefit to the qSOFA scoring system in predicting sepsis-related in-hospital mortality, ICU admission, and discharge home.

## Introduction

Sepsis remains a leading cause of mortality, with a mortality rate of over 210,000 people per year just in the United States alone [1]. There has been a 52.8% reduction in sepsis-related mortality from 1990 to 2017 largely due to the efforts of the Surviving Sepsis Campaign to identify and treat sepsis early [2]. Both systemic inflammatory response syndrome (SIRS) criteria and quick Sepsis-related Organ Failure Assessment (qSOFA) have been studied for the early identification of sepsis patients [3]. SIRS criteria were first utilized in 1992 with the initial Surviving Sepsis Guidelines, however, it has been criticized as lacking specificity [3]. In 2016, qSOFA was introduced as a replacement for the SIRS scoring system, however, it lacked sensitivity [3,4]. Procalcitonin has definite utility as a marker of severe systemic inflammation, infection, and sepsis [5]. Discontinuation of antibiotics for lower respiratory tract infections based on procalcitonin levels have shown to reduce the use of antibiotics without worsening adverse outcomes [6,7]. Guidelines do recommend the use of procalcitonin measurements to decrease antibiotic use [8]. Through our literature review, procalcitonin has been utilized as an adjunct to help increase the accuracy of our sepsis-related screening tools [9]. In addition, procalcitonin has also been found to predict mortality in severe sepsis patients [10]. The relationship between procalcitonin and sepsis-related scoring systems has yet to be thoroughly studied in terms of in-hospital mortality, ICU admission rates, and discharge location. Our goal was to explore this relationship in an attempt to gain an objective marker to predict those who are at high risk for prolonged hospitalizations and subsequent complications.

## Materials and methods

Methodology

Objectives

The primary objective of this study was to determine if the addition of procalcitonin to the existing SIRS and qSOFA scoring systems could improve the predictability of in-hospital sepsis-related mortality. Secondarily, we sought to determine if the addition of procalcitonin could predict the likelihood of ICU admission and discharge home.

Study Design and Location

This was a retrospective, single-center, observational study that took place from January 1, 2017, to January 1, 2019. The study was conducted at a community hospital in Las Vegas, Nevada.

Inclusion and Exclusion Criteria

All patients ≥18 years of age that presented to the hospital during the specified dates above were considered for inclusion in the study as long as they has at least one of the following documented in the chart: respiratory rate, heart rate, temperature, blood pressure, WBC, procalcitonin, and at least one sepsis-related diagnosis. Patients who were missing any of the above-aforementioned values were excluded from the study.

Design

Patients were stratified into four groups: SIRS-positive + procalcitonin >2 ng/mL (pSIRS+), SIRS-positive + procalcitonin ≤2 ng/mL (pSIRS-), qSOFA-positive + procalcitonin >2 ng/mL (pqSOFA+), and qSOFA-positive + procalcitonin ≤2 ng/mL (pqSOFA-). The variables used to define a positive SIRS score were ≥2 of the following criteria: respiratory rate >20 breaths/minute, heart rate >90 beats/minute, temperature >38°C or <36°C, and/or a leukocytosis >12×10^9^ cells/L or <4×10^9^ cells/L. The variables used to define a positive qSOFA were ≥2 of the following criteria: respiratory rate ≥22 breaths/minute, systolic blood pressure ≤100 mmHg, and/or a diagnosis of altered mental status. (Refer to Table 4 in the Appendices for different diagnoses used to classify patients of having altered mental status).

Statistical analysis

Mortality was analyzed by chi-square testing using Statistical Analysis System (SAS) when comparing pSIRS+, pSIRS-, pqSOFA+, and pqSOFA-. ICU admission and discharge home were analyzed with Fisher’s exact testing using SAS between the four groups.

## Results

A total of 933 total patients were included from January 1, 2017 to January 1, 2019 after inclusion and exclusion criteria were applied. We observed an overall mortality rate of 21.22% (198/933). The overall average length of stay was 8.3 days. 56.16% (524/933) of these patients had at least one ICU admission during their hospitalization and 29.58% (276/933) were discharged home. 96.36% (899/933) were SIRS-positive while 32.80% (306/933) were qSOFA-positive.

When comparing patient characteristics between those alive at discharge and those who died during their hospitalization, there was a predominance of older males who had an ICU stay during their hospitalization (Table 1).

**Table 1:** Demographic Information

Patient characteristics	Alive at discharge	Died during hospitalization
Median age	64	72
Age >65	52%	70%
Male	48%	56%
Female	52%	44%
ICU stay	48%	86%
Diabetes mellitus	18%	20%
Hypertension	71%	73%
Pneumonia	62%	74%
End-stage renal disease	8%	9%

Comorbidities like end-stage renal disease, diabetes mellitus, and hypertension were similar between groups; however, pneumonia was 12% higher in those that died during their hospitalization. 74% of those that died during their hospitalization had pneumonia compared to 62% of those that were alive at discharge.

Primary and secondary outcomes

The addition of procalcitonin >2 ng/mL improved the ability to predict in-hospital mortality in patients who were SIRS-positive: 31.89% vs 16.15% (P <0.0001), respectively, (Table 2) a difference of 15.74%. pSIRS+ also had a higher ICU admission rate compared to pSIRS- (76.16% vs 47.40%, respectively, P <0.0001, a difference of 28.76%) as well as a much lower discharge to home rate (19.20% vs 34.90%, respectively, P <0.0001, a difference of 15.70%). In general, the addition of procalcitonin to qSOFA did not show statistically significant results but tended to align with our SIRS data, albeit not as dramatically. There was a slight improvement in mortality prediction (41.94% vs 36.42%, P = 0.323, a difference of 5.52%), ICU admissions (92.90% vs 89.40%, P = 0.281, a difference of 3.50%), and a lower discharge to home rate (5.81% vs 9.27%, P = 0.250, a difference of 3.46%). pSIRS+ was more specific (70.07% vs 4.35%, a difference of 65.72%) and had a higher positive predictive value (PPV) (31.89% vs 21.80%, a difference of 10.09%) in determining in-hospital mortality compared to pSIRS-. pSIRS- was more sensitive (98.99% vs 52.02%, a difference of 46.97%) and had a higher negative predictive value (NPV) (94.12% vs 84.43%, a difference of 9.69%) in determining in-hospital mortality compared to pSIRS+. The qSOFA data tended to show a similar trend to our SIRS data; however, the changes were not as drastic. Refer to Table 3 for further details.

**Table 2:** Comparison of SIRS and qSOFA with the addition of procalcitonin in regards to primary and secondary outcomes SIRS: systemic inflammatory response syndrome; qSOFA: quick Sepsis-related Organ Failure Assessment; pSIRS+: SIRS positive + procalcitonin >2 ng/mL; pSIRS-: SIRS positive + procalcitonin ≤2 ng/mL; pqSOFA+: qSOFA positive + procalcitonin >2 ng/mL; pqSOFA-: qSOFA positive + procalcitonin ≤2 ng/mL

	pSIRS-	pSIRS+	Difference between pSIRS+ and pSIRS+	P value
Mortality	93/576 (16.15%)	103/323 (31.89%)	15.74%	<0.0001
ICU admission	273/576 (47.40%)	246/323 (76.16%)	28.76%	<0.0001
Discharged home	201/576 (34.90%)	62/323 (19.20%)	15.70%	<0.0001
	pqSOFA-	pqSOFA+	Difference between pqSOFA+ and pqSOFA-	P value
Mortality	55/151 (36.42%)	65/155 (41.94%)	5.52%	0.323
ICU admission	135/151 (89.40%)	144/155 (92.90%)	3.50%	0.281
Discharged home	14/151 (9.27%)	9/155 (5.81%)	-3.46%	0.250

**Table 3:** Sensitivity, specificity, positive predictive value, and negative predictive value for in-hospital mortality for qSOFA with procalcitonin ≤2 ng/mL, qSOFA with procalcitonin >2 ng/mL, SIRS with procalcitonin ≤2 ng/mL, and SIRS with procalcitonin >2 ng/mL CI: confidence interval; PPV: positive predictive value; NPV: negative predictive value; SIRS: systemic inflammatory response syndrome; qSOFA: quick Sepsis-related Organ Failure Assessment; pSIRS-: SIRS-positive and procalcitonin ≤ 2 ng/mL; pSIRS+: SIRS-positive and procalcitonin >2 ng/mL; pqSOFA-: qSOFA-positive and procalcitonin ≤2 ng/mL; pqSOFA+: qSOFA-positive and procalcitonin >2 ng/mL

	Sensitivity% (95% CI)	Specificity% (95% CI)	PPV% (95%CI)	NPV% (95%CI)
pSIRS-	98.99 (96.40-99.88)	4.35 (3.00-6.09)	21.80 (21.45-22.16)	94.12 (79.46-98.51)
pSIRS+	52.02 (44.82-59.15)	70.07 (13566.61-73.36)	31.89 (28.24-35.77)	84.43 (82.31-86.33)
pqSOFA-	60.61 (53.43-67.46)	74.69 (71.39-77.80)	39.22 (35.30-43.27)	87.56 (85.49-89.37)
pqSOFA+	32.83 (26.34-39.84)	87.76 (85.16-90.04)	41.94 (35.36-48.81)	82.90 (81.42-84.29)

## Discussion

Mortality

The overall mortality rate of our study was 21.22% (198/933) with 56.16% (524/933) of our participants having stayed in the ICU during their hospitalization. According to National Center for health statistics, the septicemia hospital death rate increased by 17% from 2000 to 2010 [11]. The number of inpatients who died in the hospital with a first-listed diagnosis of septicemia also increased-it tripled from 45,000 in 2000 to 132,000 in 2010 [11]. Since our primary goal was to determine if the addition of procalcitonin to the existing SIRS and qSOFA scoring systems could improve the predictability of in-hospital sepsis-related mortality, our inclusion criteria selected for septic patients. This effect was intentional in order to test our hypothesis. Of note, this data was collected pre-COVID-19 pandemic.

Primary and secondary outcomes

The goal of the initial SIRS scoring system in 1991 was to create a highly sensitive set of parameters in order to establish a time-sensitive approach to the diagnosis of sepsis and initiation of early intervention to reduce morbidity and mortality. However, by doing so, the unavoidable corollary was a lack of specificity, which prompted the development of the qSOFA scoring system in 2016 [12,13]. Although the new scoring system was more specific for sepsis and was a better predictor of in-hospital mortality and organ dysfunction in non-ICU and ER patients, it too had its pitfalls-a low sensitivity [14-18]. This prompted the search for a biomarker that could potentially assist in our quest to find a screening tool that was both sensitive and specific. Procalcitonin is one such biomarker that has been shown to rise rapidly in lieu of bacterial infection making it an ideal biomarker for improved diagnostic and prognostic value [19-24]. With this framework and background, our data can be better interpreted.

In regards to the SIRS scoring system, the addition of procalcitonin >2 ng/mL proved to be a statistically significant tool in determining in-hospital mortality, ICU admission, and discharge home when compared to procalcitonin ≤2 ng/mL. Our data is consistent with the procalcitonin literature as stated earlier and improved SIRS prognostic value, which was inherently poor. Our results demonstrate the utility of procalcitonin in aiding clinicians in determining who is most at-risk for death during their hospitalization. This is a critical tool in guiding clinicians’ decision-making as it can trigger earlier discussions about the goals of care. Palliative consultations before day 5 have been shown to reduce the length of stay and the financial burden on the healthcare system [25-29]. In addition, this can also initiate more aggressive management from admission and identify those most at risk for ICU admission.

The addition of procalcitonin to qSOFA did not show a statistically significant difference; however, the data still tended to correlate with our SIRS data. This is not entirely surprising as the utility of qSOFA is mainly a prognostic tool for morbidity and mortality. Therefore, the addition of another prognostic tool like procalcitonin would not presumably add a significant contribution. With that being said, we are not entirely certain this was the sole case. It could be possible that our information-gathering process itself (we utilized different diagnoses for altered mental status, refer to Table 4 in the appendices) may have underestimated the number of patients meeting the two-out-of-three criteria for a positive qSOFA score leading to an insignificant p-value.

Our study has multiple limitations. Firstly, patients with qSOFA scores might have been under or overestimated as only discharge diagnoses similar to altered mental status were used to classify patients into qSOFA. Secondly, only patients who had procalcitonin levels drawn were included in the study and so ICU patients who had no procalcitonin level were not included in the study. Thirdly; this being an observational study, it might have selection bias as only patients admitted with sepsis discharge diagnosis were included in the study. Even after multiple limitations, our study gives a unique insight into improving the SIRS score for predicting sepsis mortality.

Our results demonstrate that the utility of procalcitonin along with SIRS necessitates further prospective studies to validate its use as a predictor of clinical outcomes in sepsis.

## Conclusions

We conclude that procalcitonin added utility to the SIRS scoring system in predicting sepsis-related in-hospital mortality, ICU admission, and discharge home. This can assist clinicians in identifying patients with higher mortality and help triage resources appropriately. Procalcitonin did not add statistically significant benefit to the qSOFA scoring system in predicting sepsis-related in-hospital mortality, ICU admission, and discharge home.

## Appendices

**Table 4:** Discharge diagnoses used for altered mental status to be included in qSOFA score NIHSS score: National Institutes of Health Stroke Scale Score; qSOFA: quick Sequential Organ Failure Assessment

Diagnoses used to classify patients as having altered mental status		
Altered mental status	Wernicke’s encephalopathy	Vascular dementia without behavioral disturbance
Vascular dementia with behavioral disturbance	Dementia in other diseases classified elsewhere without behavioral disturbance	Dementia in other diseases classified elsewhere with behavioral disturbance
Unspecified dementia without behavioral disturbance	Unspecified dementia with behavioral disturbance	Amnestic disorder due to known psychological condition
Delirium due to known physiological condition	Post-concussion syndrome	Alcohol abuse with intoxication, uncomplicated
Alcohol abuse with intoxication, unspecified	Alcohol dependence with intoxication delirium	Alcohol dependence with withdrawal delirium
Alcohol use, unspecified with alcohol-induced persisting amnestic disorder	Other psychoactive substance use, unspecified with psychoactive substance-induced psychotic disorder, unspecified	Paranoid schizophrenia
Schizophrenia, unspecified	Delusional disorders	Brief psychotic disorder
Unspecified psychosis not due to a substance or known physiological condition	Bacterial meningitis, unspecified	Non-pyogenic meningitis
Meningitis, unspecified	Encephalitis and encephalomyelitis, unspecified	Encephalitis and encephalomyelitis in diseases classified elsewhere
Intracranial abscess and granuloma	Intracranial and intraspinal phlebitis and thrombophlebitis	Huntington’s disease
Parkinson’s disease	Other Alzheimer’s disease	Alzheimer’s disease, unspecified
Other frontotemporal dementia	Degeneration of nervous system due to alcohol	Dementia with Lewy bodies
Mild cognitive impairment, so stated	Degenerative disease of nervous system, unspecified	Other seizures
Other generalized epilepsy and epileptic syndromes, not intractable, with status epilepticus	Localization-related (focal) (partial) symptomatic epilepsy and epileptic syndromes with complex partial seizures, not intractable, with status epilepticus	Other generalized epilepsy and epileptic syndromes, not intractable, without status epilepticus
Epilepsy, unspecified, not intractable, with status epilepticus	Epilepsy, unspecified, not intractable, without status epilepticus	Transient global amnesia
Transient cerebral ischemic attack, unspecified	Communicating hydrocephalus	Obstructive hydrocephalus
(Idiopathic) normal pressure hydrocephalus	Hydrocephalus in diseases classified elsewhere	Hydrocephalus, unspecified
Toxic encephalopathy	Anoxic brain damage, not elsewhere classified	Encephalopathy, unspecified
Metabolic encephalopathy	Other encephalopathy	Compression of brain
Cerebral edema	Rain death	Disorder of brain, unspecified
Other disorders of meninges, not elsewhere classified	Hypertensive emergency	Hypertensive crisis, unspecified
Cardiac arrest due to underlying cardiac condition	Cardiac arrest due to other underlying condition	Cardiac arrest, cause unspecified
Nontraumatic subarachnoid hemorrhage, unspecified	Nontraumatic intracerebral hemorrhage in hemisphere, subcortical	Nontraumatic intracerebral hemorrhage in hemisphere, cortical
Nontraumatic intracerebral hemorrhage in brain stem	Nontraumatic intracerebral hemorrhage in cerebellum	Nontraumatic intracerebral hemorrhage, intraventricular
Nontraumatic intracerebral hemorrhage, multiple localized	Nontraumatic intracerebral hemorrhage, unspecified	Nontraumatic subdural hemorrhage, unspecified
Nontraumatic acute subdural hemorrhage	Nontraumatic chronic subdural hemorrhage	Nontraumatic intracranial hemorrhage, unspecified
Cerebral infarction due to embolism of unspecified precerebral artery	Cerebral infarction due to unspecified occlusion or stenosis of left carotid arteries	Cerebral infarction due to embolism of unspecified cerebral artery
Cerebral infarction due to embolism of right middle cerebral artery	Cerebral infarction due to embolism of left middle cerebral artery	Cerebral infarction due to embolism of bilateral anterior cerebral arteries
Cerebral infarction due to embolism of bilateral posterior cerebral arteries	Cerebral infarction due to embolism of left cerebellar artery	Cerebral infarction due to embolism of bilateral cerebellar arteries
Cerebral infarction due to embolism of other cerebral artery	Cerebral infarction due to unspecified occlusion or stenosis of right middle cerebral artery	Cerebral infarction due to unspecified occlusion or stenosis of left middle cerebral artery
Cerebral infarction due to unspecified occlusion or stenosis of left posterior cerebral artery	Cerebral infarction due to unspecified occlusion or stenosis of other cerebral artery	Other cerebral infarction
Cerebral infarction, unspecified	Occlusion and stenosis of basilar artery	Occlusion and stenosis of right carotid artery
Occlusion and stenosis of left carotid artery	Occlusion and stenosis of bilateral carotid arteries	Occlusion and stenosis of unspecified carotid artery
Occlusion and stenosis of left middle cerebral artery	Occlusion and stenosis of unspecified cerebral artery	Hypertensive encephalopathy
Cerebral ischemia	Posterior reversible encephalopathy syndrome	Unspecified symptoms and signs involving cognitive functions following cerebral infarction
Other sequelae of cerebral infarction	Acute and subacute hepatic failure with coma	NIHSS score (0-22)
Coma	Persistent vegetative state	Transient alteration of awareness
Disorientation, unspecified	Other amnesia	Altered mental status, unspecified
Other symptoms and signs involving cognitive functions and awareness	Intracranial space-occupying lesion found on diagnostic imaging of central nervous system	Traumatic subdural hemorrhage with loss of consciousness of 30 minutes or less, initial encounter
Unspecified injury of head, initial encounter	Other foreign object in bronchus causing asphyxiation, initial encounter	Other foreign object in other parts of respiratory tract causing asphyxiation, initial encounter
Food in respiratory tract, part unspecified causing asphyxiation, initial encounter	Adverse effect of insulin and oral hypoglycemic antidiabetic drugs, initial encounter	Poisoning by 4-aminophenol derivatives, intentional self-harm, initial encounter
Poisoning by other opioids, accidental (unintentional), initial encounter	Poisoning by other opioids, intentional self-harm, initial encounter	Poisoning by heroin, accidental (unintentional), initial encounter
Adverse effect of other opioids, initial encounter	Adverse effect of other synthetic narcotics, initial encounter	Poisoning by cocaine, accidental (unintentional), initial encounter
Poisoning by unspecified narcotics, accidental (unintentional), initial encounter	Adverse effect of unspecified narcotics, initial encounter	Poisoning by benzodiazepines, accidental (unintentional), initial encounter
Poisoning by benzodiazepines, intentional self-harm, initial encounter	Poisoning by benzodiazepines, undetermined, initial encounter	Adverse effect of benzodiazepines, initial encounter
Poisoning by other antiepileptic and sedative-hypnotic drugs, accidental (unintentional), initial encounter	Poisoning by other antiepileptic and sedative-hypnotic drugs, intentional self-harm, initial encounter	Adverse effect of other antiepileptic and sedative-hypnotic drugs, initial encounter
Adverse effect of unspecified antiepileptic and sedative-hypnotic drugs, initial encounter	Adverse effect of other antipsychotics and neuroleptics, initial encounter	Poisoning by amphetamines, accidental (unintentional), initial encounter
Adverse effect of amphetamines, initial encounter	Adverse effect of other parasympatholytics- anticholinergics and antimuscarinics and spasmolytics, initial encounter	Adverse effect of antineoplastic and immunosuppressive drugs, initial encounter
Adverse effect of unspecified drugs, medicaments and biological substances, initial encounter	Adverse effect of other drugs, medicaments and biological substances, initial encounter	Toxic effect of ethanol, accidental (unintentional), initial encounter
Toxic effect of unspecified alcohol, accidental (unintentional), initial encounter	Toxic effect of other organic solvents, accidental (unintentional), initial encounter	Toxic effect of other specified substances, accidental (unintentional), initial encounter
Toxic effect of unspecified substance, accidental (unintentional), initial encounter	Angioneurotic edema, initial encounter	Blood alcohol level of less than 20 mg/100 mL
Blood alcohol level of 20-39 mg/100 mL	Blood alcohol level of 100-119 mg/100 mL	Presence of alcohol in blood, level not specified
